# Task Sharing or Task Dumping: Counsellors Experiences of Delivering a Psychosocial Intervention for Mental Health Problems in South Africa

**DOI:** 10.1007/s10597-020-00734-0

**Published:** 2020-11-08

**Authors:** Y. Jacobs, B. Myers, C. van der Westhuizen, C. Brooke-Sumner, K. Sorsdahl

**Affiliations:** 1 Alcohol, Tobacco and Other Drug Research Unit, South African Medical Research Council, Cape Town, South Africa; 2 Alan J. Flisher Centre for Public Mental Health, Department of Psychiatry and Mental Health, University of Cape Town, Cape Town, South Africa; 3 Department of Psychiatry and Mental Health, University of Cape Town, Cape Town, South Africa

**Keywords:** Task-sharing, Common mental disorders, Training and supervision, South Africa

## Abstract

Given task-sharing mental health counselling to non-specialist providers is a recognised strategy to increase service capacity, ensuring that their training, supervision, and support needs are met is necessary to facilitate the sustainable delivery of a high-quality service. Using in-depth interviews, we qualitatively explored the experiences of 18 facility-based counsellors (FBCs) tasked with delivering a counselling intervention within chronic disease services offered within primary care facilities participating in the project MIND cluster randomised controlled trial. Findings show that project MIND training with a strong emphasis on role playing and skills rehearsal improved FBCs’ confidence and competence, complemented by highly structured supervision and debriefing provided by a registered counsellor, were key strategies for supporting the implementation of task-shared mental health counselling. FBCs perceived many benefits to providing mental health counselling in primary healthcare but systemic interventions are needed for sustained implementation.

## Introduction

Untreated mental disorders are highly prevalent in South Africa. The South African Stress and Health study (SASH), a nationally representative study, found that the 12-month prevalence of any mental disorder was 16.7% (Williams et al. 2008), with a life-time prevalence of 30.3% (Stein et al. 2008). Anxiety disorders had the highest lifetime prevalence (15.8%), followed by alcohol use disorders (11.4%) and mood disorders (9.8%) (Stein et al. 2008). Despite this high prevalence of common mental disorders (CMDs), only 25% of those meeting criteria for a 12-month mental disorder received treatment (Seedat et al. 2008). This large treatment gap is similar to that found in other low-and middle-income countries (LMICs) (Demyttenaere et al. 2004).

Several factors contribute to this treatment gap in South Africa. These include structural and financing barriers, low perceived need for treatment, low mental health literacy, stigma, and systemic barriers (Ameh et al. 2017; Bruwer et al. 2011; Hugo et al. 2003; Mendenhall et al. 2014). A key system constraint is the limited availability of mental healthcare providers. Nationally, the average number of psychiatrists working in the public sector is 0.31 per 100,000 people with 0.97 psychologists per 100,000 people (Docrat et al. 2019). Consequently, few mental health services are provided on the primary care platform; services that are available focus on medication provision for people with severe mental illness, with little provision of mental health counselling for individuals with CMDs (Docrat et al. 2019).

Given the human resource constraints, the country has endorsed the World Health Organization’s (WHO) recommendation of “task sharing” mental health counselling to non-specialist providers, including facility-based counsellors (FBCs) who work within primary healthcare (PHC) facilities (Department of Health Republic of South Africa 2013). In the South African setting, FBCs are a specific cadre of community health workers trained to deliver health promotion and HIV adherence counselling services. Within this task-sharing framework, the WHO has identified two approaches: either mobilizing the human resources currently available within PHC services (such as FBCs) by expanding their current roles to include counselling (termed the “*designated approach*”) or re-distributing funding to allow for the employment of additional counsellors (termed the “*dedicated approach*”) (World Health Organisation 2007).

In South Africa, and other LMICs, it remains to be shown which human resource configuration is effective, feasible, and acceptable for delivering counselling. Project MIND is investigating the relative effectiveness of the designated and dedicated approaches to integrating a FBC-delivered intervention for depression and alcohol use disorders into chronic disease services (Myers et al. 2018). In addition to the clinical outcomes of this trial, information on the experiences of both dedicated and designated FBCs is needed to guide health planners’ decision making. FBCs are likely to be the frontline workers tasked with the delivery of these services; ensuring that their training, supervision, and support needs are met will help support the delivery of a high-quality services.

Concerns regarding the high levels of responsibility placed on FBCs with no formal mental health training, the degree to which they are supported in this new role, and the potential negative impact that insufficient support and training may have on both counselling outcomes and their own well-being are highlighted in the literature (Agyapong et al. 2016; Shahmalak et al. 2019). FBCs also face common challenges in PHC settings, including lack of private spaces in which to provide counselling, feeling unwelcome and marginalised from the rest of the PHC team, lack of confidence in their role, and lack of clarity on role functions (Petersen et al. 2016). Further, although counsellors may have the skills to deliver the technical content of the counselling interventions, the more nuanced micro skills such as reflecting on feelings and interpreting clients’ responses may require intensive training (Kagee 2013).

Several studies in LMICs, including South Africa, have investigated the acceptability, feasibility, and potential effectiveness of using dedicated FBCs to deliver mental health counselling at PHC facilities (Mendenhall et al. 2014; Padmanathan and De Silva 2013; Singla et al. 2017; Spedding 2014), including the Friendship Bench in Zimbabwe (Chibanda et al. 2016), the MANAS trial in India (Patel et al. 2010), and project STRIVE in South Africa (Sorsdahl et al. 2015). However, only a handful of studies from LMICs have explored the experiences of counsellors responsible for the delivery of these interventions (Shahmalak et al. 2019). These studies suggest that counsellors are generally highly motivated, experience growth from training and supervision, and apply concepts from the intervention to address difficulties in their own lives (Munodawafa et al. 2017; Shahmalak et al. 2019. While these studies have conducted an in-depth exploration of the FBCs’ experiences of training and supervision (Barnett et al. 2018), the potential differences in the experiences of *designated* or *dedicated* counsellors remains unknown. An understanding of FBCs’ perceptions of the most beneficial aspects of training and supervision (e.g. content, intensity, duration) can guide the development of training and supervision models for the scale up of mental health counselling in these settings. Comparing the experiences of designated and dedicated counsellors is also relevant since they are likely to face different systemic challenges to delivering counselling. Implementation strategies required to support the scale up of dedicated versus dedicated approaches may therefore need to vary.

This paper helps provide this information through exploring the experiences of both dedicated and designated FBCs tasked with delivering the project MIND counselling intervention within primary care chronic disease services in the Western Cape Province of South Africa. More specifically, the study aims to explore FBCs’ perceptions of (1) the training they received to deliver the MIND intervention, (2) difficulties in implementing the intervention within PHC services and (3) their perceptions of the supervision they received to support intervention delivery.

## Methods

This manuscript complies with the Consolidated Criteria for Reporting Qualitative Research (COREQ) (Tong et al. 2007).

### Study Context, Intervention, and Implementation

This qualitative study interviewed FBCs providing the project MIND intervention in 16 PHC facilities assigned to one of the study’s intervention arms. Project MIND is a cluster randomised trial in which 24 primary care facilities were randomly allocated to a dedicated, designated or treatment as usual arm (Myers et al. 2018). In the eight facilities allocated to the dedicated arm, a FBC was added to the chronic disease team and trained to provide a three-session blended Motivational Interviewing and Problem-solving Therapy (MI-PST) intervention for patients who screened at-risk for a CMD, specifically depression or problem alcohol use (Myers et al. 2018). Each facility has a free HIV and chronic disease management unit. These units are generally staffed by a medical doctor, professional nurses and HIV adherence counsellors or health promoters who provide adherence counselling and/or lifestyle advice.

In the eight facilities allocated to the designated arm, a FBC was identified from the existing pool of FBCs at that facility and trained to provide the MI-PST intervention in addition to their usual adherence counselling duties. An apprenticeship model of supervision was used (Murray et al. 2011), where both “dedicated”, and “designated” FBCs received regular supervision, debriefing, and in-service training by a registered psychological counsellor with experience in delivering MI-PST. This model of training and supervision was identical for both dedicated and designated counsellors. This psychological counsellor followed a structured approach to supervision and received supervision and support from a psychologist. Table 1 describes the project MIND intervention, training, and supervision model.

**Table 1:** Components of the project MIND intervention package

Theoretical framework (intervention)	Lazarus and Folkman’s coping theory (Lazarus and Folkman 1984) informs the problem-solving therapy (PST) approach: problem-solving for problems that can be solved and emotion-focused coping for problems that can’t be solved.
Delivering agents	Facility-based counsellors (FBCs) who had completed high school and had been trained to provide HIV adherence counselling: *Dedicated intervention arm*: FBCs added to the existing chronic disease team deliver the intervention. *Designated intervention arm*: FBCs currently part of chronic disease team deliver the intervention.
Structure of intervention package	3 sessions of blended motivational interviewing/problem-solving therapy (MI-PST), with optional booster, to be delivered weekly. Participants had a 6-week window to complete all sessions before timing out of the intervention.
Structure of sessions	
Session #1	Provide feedback on mental health assessment. Increase knowledge of depression and alcohol use and their impact on the course of HIV and diabetes. Identify a behaviour to modify and use motivational interviewing (MI) techniques to build rapport and deve lop readiness to change. Develop a change plan. Describe Take Home Activity #1.
Session #2	Patient check-in using MI. Review activities from session 1. Build the rationale for PST. Teach the steps of PST. Conduct 2 problem-busting sessions. Describe Take Home Activity #2.
Session #3	Patient check-in using MI. Review activities from session 2. Coping with negative thoughts : Explain how to cope with problems that are not important. Advance process of acceptance : teach how to deal with problems that are important and cannot be solved. Conduct a problem-busting session.
Booster Session	Patient check-in using MI. Review of previous activities. Conduct a problem-busting session.
FBC training	
Characteristics of trainers	Two registered psychological counsellors (RCs) with a 4-year Psychology Honours-level qualification and registered with the Health Professions Council of South Africa (HPCSA). 3 years past experience in delivering the MI-PST intervention and training healthcare workers. Training oversight and support were provided to RCs by a psychologist with a doctoral qualification and 10 years previous experience in developing MI-PST intervention models and training.
Structure and format of training	40 hours of formal training (the equivalent of five working days). Mixture of didactic teaching and experiential group activities including skills rehearsal exercises and role plays. Counselling proficiency assessed during role plays using a counselling fidelity checklist. Knowledge, attitudes, beliefs and practices around counselling for CMDs assessed pre-and post-training. Booster training involving MI-PST counselling skills provided in-service to all FBCs one month after completion of formal training.
Training content	Information on common mental disorders (CMDs), diabetes and HIV. Basic counselling communication skills training. Screening patients for CMDs training. MI skills training. PST skills training. Delivery of the MI-PST intervention. Ethics: managing distressed participants and referral for additional care.
Characteristics of supervisor	5 years previous counselling experience in cognitive-behavioural therapy-based brief interventions. 3 years previous experience in delivering MI-PST and training healthcare workers. Conduct guided by the professional standards and ethics–Professional Board for Psychology and the HPCSA.
Structure of supervision and debriefing	In-person or telephonic individual supervision and debriefing conducted once a week. Telephonic supervision and debriefing used during community unrest, gang violence or geographically distant healthcare facilities. Brief communication via text or WhatsApp messaging to address challenges in real-time in between weekly scheduled supervision and debriefing sessions. Supervision and debriefing lasted up to an hour per session and structured as follows: Debriefing: brief check-in, followed by reflection on recent experiences at work and/or home, how these experiences were dealt with emotionally and practically (coping) and identifying opportunities for growth. Clinical supervision: FBCs presented new cases and/or discussed patient progress including feedback, suggestions or recommendations by supervisor where needed. Addressing challenges: logistical and counselling delivery challenges were discussed, and solutions brainstormed. Counselling fidelity feedback: FBCs provided with structured feedback on their counselling proficiency using a counselling fidelity checklist (audio tapes of counselling sessions assessed prior to supervision by supervisor). Brief skills rehearsal exercises or role playing used to improve and solidify counselling aspects with average to low scores on the fidelity checklist. RC trained to use a structured approach to supervision.
Supervisor training and support	Weekly in-person or telephonic supervision provided by a psychologist who assessed adherence to the supervision approach and discussed ways of overcoming any logistical and systemic challenges to provision of supervision and debriefing. Weekly in-person or telephonic debriefing provided by a psychologist, which included reflecting on recent experiences at work and/or home, how these experiences were dealt with emotionally and practically (coping) and identifying opportunities for growth.

### Participants

All dedicated and designated counsellors were eligible for the study after they had been delivering the intervention for at least three months. Twenty-nine FBCs who delivered the MIND intervention in 16 PHC facilities assigned to one of the intervention arms were invited to participate. A total of 18 FBCs agreed to be interviewed, and 11 declined the invitation due to work responsibilities. The FBCs who declined to participate were dispersed across a range of facilities and therefore should not have introduced a systematic bias to responses. Among the counsellors who participated, 8 (44%) were from the *Dedicated* arm and 10 (56%) from the *Designated* arm. More than half of the FBCs were female (72%) reflecting the general demographic profile of FBCs across PHC facilities, and with an average age of 40 years (SD = 9.1). In terms of previous work experience, the majority of designated FBCs started working for non-government organisations as community health workers (CHWs) before progressing to work as HIV adherence counsellors located within PHC facilities. All the FBCs had completed high school (12 years of education) and training in HIV adherence counselling. This training focused on sharing HIV-specific health promotion information and providing adherence support. Dedicated FBCs had similar work experience but were unemployed before being employed to deliver the MIND intervention.

### Procedure

An experienced qualitative interviewer, with a master’s level qualification and independent of the study team, approached each FBC in-person after their weekly supervision session and invited them to participate. The interviewer was not known to the FBCs before this study. The purpose and aims of the study were described including potential risks and benefits, privacy and confidentiality, and the rights of participants to refuse participation. Written informed consent was obtained from those willing to participate and a mutually beneficial time was scheduled to conduct the interviews. Information pertaining to whether the FBC participated in the interviews was not shared with Project MIND staff. All interviews were conducted in English and lasted approximately 45 min to an hour. Interviews followed a semi-structured guide that included questions pertaining to FBCs’ perceptions of training, barriers and facilitators to providing the intervention within the health facility, and their perceptions of supervision and supportive debriefing. All interviews were digitally recorded and took place in the private counselling room in the PHC facility at which the FBC was located. Participants were given a grocery voucher to thank them for their time and participation. Recordings were transcribed verbatim by a professional transcriber. All transcriptions were anonymized and the identities of those who refused participation withheld. Ethical approval was obtained from both the University of Cape Town (HREC REF 753/2017) and the South African Medical Research Council (EC 004-2/2015) human research ethics committees. The authors have no known conflicts of interest and all authors certify responsibility for the manuscript.

### Data Analysis

NVivo 12 software facilitated data analysis. We used the framework approach to analyse the transcripts (Ritchie and Spencer 1994). This approach allows themes to be explored in relation to the research questions, for new themes and minority perspectives to emerge from the data. The first author conducted the initial familiarization with the data through review, initial coding and identification of major themes. The first author and a second researcher coded the first five interviews independently. Both coders met regularly to review and refine the codes for consensus. Data saturation was reached after coding twelve transcripts, with the remainder coded according to the agreed upon themes. A code breaker was not needed to resolve coding discrepancies. After the themes were identified from the data, member checks were done by the first author. FBCs were approached in-person and presented with the themes and an opportunity to comment on whether the data and the interpretation thereof were accurately captured. No new information emerged from member checking.

## Results

Results are presented according to the three major themes that emerged: (1) counsellors’ perceptions of the project MIND training; (2) counsellors’ perceptions of the clinical supervision and debriefing provided by project MIND; and (3) perceived barriers and facilitators to the implementation of the project MIND counselling.

### Counsellors’ Perceptions of the Project MIND Training

Counsellors described how the project MIND training supported implementation of task-shared mental health counselling. Training was well received by all FBCs who perceived several benefits in terms of building their professional counselling skills. In particular, designated counsellors highlighted their improved communication skills (such as the use of active listening, open-ended questions, and reflections) and understanding of psychosocial problems. They described applying these to their usual adherence counselling practice, enhancing their counselling relationships and improving counselling quality.

“*It *[project MIND training]* was different because mainly at *[organisation providing adherence counselling training]* you talk about HIV and so, it has nothing to do with a person’s psychological state *…* this one is psychological all the way because it is a person’s problems. So that is the difference.*” (ID06, designated counsellor).

“*If we have a defaulter* [patient who stopped taking ARVs] *we can ask them what is the reason, why did you not come, what made you feel that way*…*which I did not do before because we did not go on the *[project MIND]* training *… *we did not know about this *…” (ID11, designated counsellor).

Most FBCs described how being trained in MI-PST benefitted them personally. After seeing how the problem-solving method helped their patients, some of these counsellors applied the problem-solving method to resolve their own challenges and assist those around them (e.g. problematic alcohol use among family members).

“*It benefitted me because my husband also drinks* [alcohol], *so … I have given him my [intervention] book. And then it was very helpful, and he was interested* …” (ID01, designated counsellor).

The FBCs emphasised that the role-playing and skills rehearsal enhanced their confidence in their ability to deliver the MIND intervention. According to these counsellors, the skills rehearsal aspect of training provided them with opportunities to rehearse their new counselling skills while simultaneously familiarising themselves with the manualised nature of the intervention:

“*I learned so much about role plays so that we can familiarise how the intervention should be like* …* from session one to session three*.” (ID08, dedicated counsellor).

Both designated and dedicated FBCs recommended ways to improve training. These included incorporating even more opportunities for role-play and skills rehearsal and adding psychoeducational and counselling content related to other drugs of abuse. Some FBCs described struggling to maintain concentration during training and they were not accustomed to the intensity of each training day (6–7 h per day). These FBCs felt that they would benefit more from fewer hours of training per day and extending the number of days of training:

“*The hours were too long. I mean too long hours can also put a person to like* … *get tired.*” (ID17, dedicated counsellor).

### Counsellors’ Perceptions of Clinical Supervision and Debriefing

Within project MIND, the provision of regular clinical supervision and debriefing to FBCs emerged as another important strategy for supporting the implementation of task-shared mental health counselling.

All FBCs reported being largely satisfied with the supervision and debriefing they received, emphasizing its role in their professional development and for ensuring quality of counselling. Weekly supervision and debriefing enhanced both their counselling skills and their confidence in their ability to deliver the intervention. They described valuing the opportunity that supervision afforded to discuss patient progress, assess counselling performance, and identify ways of improving their practice.

“*I think for me supervision is important because you can get help with whatever case or any difficult situations that we are facing and also to know where to work on. It is also nice to hear that, oh, you are doing good or you can work on that or maybe do not use that and so on. So that really, really, really helps a person*.” (ID03, designated counsellor).

FBCs also valued the regular debriefing they received as part of their weekly supervision session, particularly in relation to prioritising self-care and preventing burnout. Regular opportunities for debriefing created a “safe space” to discuss difficult patient cases, personal issues that may be impacting on their counselling, issues around self-care, and strategies for coping with stress:

“*It is very important, because before I used to take on the whole world. Now, I know that as I care for my client, I must care for myself, because if I do not care for myself, I am going to burn out.*” (ID18, designated counsellor).

When asked, FBCs identified several factors that either supported or hindered the degree to which they could engage and benefit from supervision and debriefing. With regards to the format and delivery of clinical supervision and debriefing, FBCs found the role-playing exercises conducted during supervision particularly beneficial. During these role-plays, the FBC and supervisor would re-enact challenging counsellor-patient interactions. The counsellor would assume the role of the patient and the supervisor the role of the counsellor. These role plays provided an opportunity to reflect on alternative approaches to problems, enabling the FBCs to reflect on past situations and practice managing them differently while preparing them to deal with such scenarios more effectively going forward.

“*We do a bit of role-play also … so that is the one thing that stands out for me … when you put yourself in a client’s position … or you change roles, gives you a bit of an idea of what you must do.*” (ID03, designated counsellor).

FBCs described a good working relationship with the counselling supervisor as critical, describing the need for a supervisor that was trustworthy, respectful, and non-judgmental. FBCs also viewed the availability of the supervisor outside of formal supervision times as key for supporting them in their new role. They described *“debriefing on-the-go”* as key to the success of the programme:

“*He* [counsellor supervisor] *is always there to support, even via WhatsApp. I can ask him anything, any time he will respond swiftly.*” (ID15, dedicated counsellor).

FBCs provided suggestions for how the project MIND approach to supervision and debriefing could be strengthened including longer supervision sessions and more time with the supervisor, particularly during the first few weeks of their new role. As one FBC noted, “…* when he leaves, I just feel that, oh, I needed one more hour.*” (ID04, dedicated counsellor). Some expressed the desire for occasional group supervision and debriefing to help them feel connected and part of a community of practice where they could share their experiences and learn from each other.

“*If they can have mentoring [supervision and debriefing] where all the counsellors get together and then they debrief* … *with different cases and how they struggle and how do they cope.*” (ID06, designated counsellor).

Logistical challenges to the uptake of supervision and debriefing were identified. These included a lack of private space and difficulties adhering to the supervision schedule. Dedicated counsellors described challenges relating to lack of private space for clinical supervision and debriefing but did not describe difficulties adhering to the supervision schedule. In contrast, space was less of an issue for most designated counsellors. However, all the designated counsellors experienced difficulties adhering to the supervision schedule – largely due to competing demands on their time from the other work responsibilities. The PHC facilities in which these counsellors worked had very high patient volumes which resulted in the designated counsellors having to prioritise attending to those patients at the expense of supervision and debriefing.

“… *and when we have a problem that we cannot have that supervision, we will tell [the counsellor supervisor] that the clinic is busy today and then this and this and this is going on … so the times of supervision … it is not going to work*.” (IDO6, designated counsellor).

### Perceived Barriers and Facilitators to the Implementation of the Project MIND Intervention

FBCs noted the length and complexity of the intervention relative to usual care (e.g. HIV adherence counselling). Most designated counsellors reported that “*It is very helpful, but it is too long*”. These FBCs described their feelings of concern about the many other patients waiting to be seen while they were spending up to an hour counselling a single project MIND patient.

However, despite the perceived length and complexity of the intervention, all the FBCs perceived the project MIND intervention as beneficial for their patients, extending beyond the adherence counselling currently available within chronic disease services. They thought the project MIND intervention offered a “relative advantage” over this counselling by helping patients address their underlying psychosocial problems that impact on their medication adherence which standard adherence counselling did not do. FBCs’ beliefs in the advantage of this intervention were underscored by their observations of improvements in their patients’ outward physical appearance and perceptions that patients were taking more responsibility for their health after completing the project MIND counselling programme.

“*The nicest part is when you can actually see the changes. When the patient comes back for the follow-up and you can see the difference before and now after.*” (ID13, designated counsellor).

“*What I like about this, implementing this intervention is that it—for the patient that was not compliant, is compliant now. I think our rate of defaulters, the patients that we have seen has decreased.*” (ID18, designated counsellor).

Designated counsellors expressed concerns about the degree to which they could align the delivery of mental health counselling with their current workflow. They described competing task demands (including the management of chronic clubs which involves health promotion, provision of HIV testing and counselling, and other administrative duties) and uncertainty about how to reorganise their work schedule to accommodate MIND counselling. Several FBCs felt overwhelmed by these multiple task demands and needed help to prioritise their other work demands so that they could perform their duties optimally.

“*At the beginning it was fine but as time went by it was such a lot of people we had to see and we had to see our work as well. So, it was tiring, because an hour to sit with a person is a lot when you know my colleagues need to see now maybe 20 or 30 more people because I am busy in this project.*” (ID11, designated counsellor).

By contrast, dedicated counsellors did not report the anxiety around competing task demands. Given their primary work task was mental health counselling, they were able to focus on counselling without feeling pressured to perform other tasks. When they were not busy counselling patients, they described offering their assistance to others in the facility such as “*going to the reception to help them with the folders*” or helping “*if there is a doctor or the nurse that needs a translator*”.  Although more salient for designated FBCs, both types of FBCs explained how their role was assigned a low status within the healthcare facilities. Counsellors perceived their lack of status and authority as hindering their efforts to negotiate for private and a confidential space within which to deliver counselling. This was evident in the many disruptions that would occur during the counselling sessions when healthcare staff would often enter the room with a query or request. Both designated and dedicated FBCs felt that these interruptions reflected the low value that other health workers placed on their work, which contributed to feelings of marginalisation.

“…*Sometimes they [nurses] just say, ‘oh, this is the lay counsellor’, you know, you are just* [irrelevant]”. (ID18, designated counsellor).

For a subgroup of FBCs, these feelings of being undervalued were reinforced by low levels of remuneration. In South Africa, FBCs are paid a stipend that is just above minimum wage. According to these FBCs, low remuneration decreased their motivation to provide the counselling service. Designated counsellors were particularly frustrated as their scope of work had increased without any adjustment to their remuneration. Many described situations where they felt that tasks were being ‘dumped’ on them without any recognition or reward for the work they do.

“…*we do not want to sound like [we] always wants recognition in the form of finance, but that would also help. Because I mean, your load is increasing, your targets are increasing, but money wise it is not increasing.*” (ID13, designated counsellor).

FBCs thought that other healthcare staff within the facility would benefit from mental health training to enhance their understanding of counselling and its benefits for patients, the facility, and the wider community. FBCs hoped that this additional training would lead to greater recognition of their role in the future.

## Discussion

In South Africa, like other LMICs, the implementation and scale up of task-shared mental health counselling within PHC facilities requires an FBC workforce to be trained, supervised and supported to ensure they are confident, sufficiently skilled and empowered to take on this role. Yet only a handful of studies have explored FBCs’ experiences of delivering mental health counselling and even fewer have examined FBCs’ experiences of and preferences for mental health training, supervision and implementation support (Shahmalak et al. 2019). This study provides initial insights into FBCs’ experiences of the training and supervision they received in the project MIND trial. We have presented areas where support could be modified and enhanced. Findings suggest that FBCs: (1) benefitted personally and professionally from the mental health training, but would have preferred lower intensity training over a longer period of time; (2) felt regular individual supervision and debriefing improved counselling quality and provided emotional support which could be enhanced through the addition of peer group supervision; (3) and were willing to continue with counselling implementation given its perceived benefits for patients. Several factors related to the complexity of integration of this innovation into PHC facilities and motivation of counsellors were also identified.

Both designated and dedicated counsellors considered the training they received through project MIND acceptable and relevant for capacitating them to provide mental health counselling. In keeping with earlier studies (Munodawafa et al. 2017), they described professional growth and personal benefits from participating in this training. According to these counsellors, the psychoeducation content enhanced FBCs’ mental health literacy and understanding of potential psychosocial difficulties that patients may experience. MI training improved their communication skills which impacted positively on the counsellor-patient therapeutic alliance, and the PST content equipped them with evidence-based strategies for assisting patients. These FBCs viewed the skills rehearsal and feedback activities as the most valuable of the project MIND training activities. Even though we had significantly increased the amount of time spent on skills rehearsal from the pilot phase of project MIND (Myers et al. 2019a, b), several FBCs thought that additional opportunities for skills rehearsal would have enhanced their confidence in their ability to deliver the intervention. There is growing evidence of the benefits of skills rehearsal activities relative to didactic training for specialist mental health workers (Bearman et al. 2013; Beidas and Kendall 2010; Fairburn and Cooper 2011) and emerging evidence that skills rehearsal activities may be especially helpful for establishing counselling confidence and competence when training non-specialist providers with low education levels (Barnett et al. 2018; Murray et al. 2011). Our findings suggest that it is also important to ensure that the structure and format of training allows for the counsellors to engage with the new material. Our counsellors described difficulties in concentrating for extended periods of time which affected their engagement in the training. To overcome this challenge during scale up of the project MIND intervention, they recommended reducing the duration of each training day while increasing the number of training days, thus allowing sufficient time to cover all the training material and opportunities to reflect. Taken together, these findings begin to address a gap in the global mental health literature (Barnett et al. 2018) by identifying potentially effective approaches to training FBCs in mental health counselling provision, although the extent to which these strategies are effective is yet to be established.

Study findings also provide insight into FBCs’ experiences of counselling supervision and debriefing. While FBCs were not accustomed to receiving regular, intensive supervision of their counselling practice, they quickly adjusted and found it beneficial for their professional development and counselling quality. FBCs’ openness to performance feedback may have been enhanced by project MIND’s cascade approach to supervision. Most approaches to clinical supervision in global mental health rely on specialist providers as ‘experts’ to deliver supervision, creating a power differential between supervisor and supervisees that counsellors may experience as threatening, inhibiting openness and engagement in supervision (Kemp et al. 2019). In contrast to these traditional approaches, project MIND trained a registered psychological counsellor (RC) (with a 4-year degree and experience in delivering MI-PST) to provide supervision using a standardised format, the quality of which was monitored by a psychologist. Given the FBCs reflections on what facilitated a good working relationship with the supervisor, having a RC rather than a specialist psychologist in this role may have reduced the power differential. Given the limited availability of highly trained specialists to provide supervision for task-shared counselling in LMICs (Singla et al. 2014), mentorship approaches including peer supervision have been proposed (Kemp et al. 2019). Project MIND utilized a cascade approach to supervision offering a potentially scalable alternative. For this model to be implemented at scale in South Africa, training curricula of RCs will need to expand to include a stronger emphasis on the development of supervision and debriefing skills. They will also need to be employed within the public health system with supervision of generalist counsellors integrated into their job description.

In addition, our findings suggest that intentionally incorporating debriefing into weekly supervision contributed to FBCs’ positive views of supervision and helped buffer against the stress associated with counselling in this context. While the importance of providing such support for preventing burnout and improving job satisfaction among mental health providers is well-established (Edwards et al. 2006), our findings support claims that debriefing and emotional support services are critical in the context of task-shared mental health services where counsellors do not have extensive training in managing stress and preventing secondary traumatization (Barnett et al. 2018; Upadhaya et al. 2020). As in other studies (Jain 2010; Wall et al. 2020), many of the FBCs in this study were exposed to the same environmental stressors as participants, resulting in psychological distress that was exacerbated by the provision of counselling. Our findings suggest that FBCs required significantly more emotional support than originally anticipated. FBCs described wanting more contact with their supervisor, particularly when they were still inexperienced in delivering the intervention. In this study, the supervisor was readily available to provide additional support informally through virtual means; this flexible approach to the provision of support will need to be considered when developing supervision models for implementation at scale. FBCs also recommended the addition of group supervision. This is in keeping with findings from other studies that have described the value of peer supervision for mutual support (Henry et al. 2016; Kemp et al. 2019; Wall et al. 2020). Given the challenges that FBCs described in scheduling time and finding space for individual supervision, arranging face-to-face peer group supervision is likely to be difficult in this setting. However virtual supervision using technology including WhatsApp groups and online meeting platforms may be a means of overcoming some of the distance, space and time barriers associated with participating in face-to-face meetings (Acharya et al. 2017; Swar et al. 2019).

While acknowledging the support afforded through supervision, our findings point to need for health systems change to enable the integration of mental health counselling in primary care facilities. FBCs identified the need for more implementation support from the PHC facility to help them navigate systemic challenges to integrating mental health counselling into primary care. Designated FBCs were particularly concerned about ‘task dumping’, anticipating that they would be expected to deliver mental health counselling in addition to their other tasks with no re-negotiation of their existing job descriptions and targets or additional remuneration. Concerns about FBC work demands and lack of additional capacity to provide mental health counselling have been consistently raised by other researchers in this area (Padmanathan and De Silva, 2013). Should the South African Department of Health proceed with training designated FBCs to deliver mental health counselling, their job descriptions, workload and daily targets will need to be assessed and adjusted so that they have the capacity to deliver mental health counselling with fidelity. This is in keeping with recommendations for supporting tsk-sharing of mental health interventions from other health system strengthening initiatives, such as the Emerald programme (Petersen et al. 2017).

Related to this, both dedicated and designated FBCs identified the importance of orientating managers and other health care workers to having co-located mental health counselling services within the facilities. Limited understanding of the importance of mental health counselling by other facility staff and the lack of resources (confidential space and time) required to provide adequate care as key implementation barriers to address during counselling scale up. Prior studies have also identified these as potential barriers to the implementation of mental health counselling in PHC facilities (Brooke-Sumner et al. 2019; Myers et al. 2019a, b). Strong leadership from facility managers can help staff navigate many of the resource challenges to counselling implementation (Brooke-Sumner et al. 2019). Improving mental health literacy among other staff through awareness campaigns that address the impact of untreated CMDs and the benefits of mental health counselling may also help create a facility climate conducive to counselling implementation. Practical steps forward could include in-service staff training around mental health issues (Maconick et al. 2018), mental health awareness drives, and integrating mental health promotion initiatives into workplace policies (Petersen et al. 2016).

## Limitations

There are several limitations to this study. Although the sample is largely representative of the counsellors who participated in the trial, the extent to which findings are generalizable to FBCs in other parts of South Africa or other LMICs is not known. Research that examines similarities and differences among more diverse groups of counsellors across different contexts and types of mental health interventions may produce valuable findings for guiding the development of generic training and supervision models. In addition, while we took precautions to limit social desirability bias, including having an external person contact and conduct the interviews, participants may not have felt comfortable criticising the training or supervision that they received. Further, some of the FBCs approached for an interview declined due to other commitments –our sample may have been skewed towards participants who felt the training and supervision was generally acceptable and beneficial. Finally, this study was unable to determine the level and amount of training and supervision needed for FBCs to achieve competence in counselling delivery. Future research needs to address this gap so that efforts to develop models for training and supervising FBCs are guided by evidence of what works (Barnett et al. 2018).

## Conclusion

This study contributes to the emerging evidence on training and supervision models for task-shared counselling interventions in LMIC settings. Our findings highlight the value of emphasising role play and skills rehearsal during training and suggest that counsellor competency may be enhanced through ensuring that the structure and content of training is maximised. Second, this study presents evidence of the acceptability of a cascaded approach to supervision in which supervision and debriefing is task-shifted from psychologists to registered psychological counsellors. As no differences emerged between dedicated and designated counsellors’ experiences of this approach to training and supervision, findings suggest that the models proposed here are suitable for both types of counsellors. Nonetheless, findings highlight opportunities to enhance this model of supervision through the addition of virtual peer group supervision and increasing the amount of contact with FBCs, through using technology. Finally, findings confirm that FBCs perceived many benefits to providing mental health counselling to patients with chronic diseases, but systemic interventions are needed to create a PHC facility climate conducive to the sustained implementation of mental health counselling. Unlike dedicated counsellors, designated counsellors experienced difficulties related to health managers’ expectations and targets associated with their existing job descriptions. Systems strengthening interventions, such as re-negotiating job descriptions and targets to accommodate mental health counselling and facility preparedness activities to orientate other health care workers to the introduction of co-located services is needed to ensure that designated counsellors have the time and institutional support they need to deliver counselling.
